# A conversation on building safe spaces for the LGBTQ+ community in the geosciences

**DOI:** 10.1038/s41467-021-24020-z

**Published:** 2021-06-25

## Abstract

Lesbian, gay, bisexual, transgender and queer (LGBTQ+) scientists are an invisible minority that still faces harassment and discrimination. Fostering safe, designated LGBTQ+ environments is a way for the community to connect with each other and raise awareness. In honor of Pride Month (June 2021), Dr. Keisling (postdoctoral fellow at the Lamont-Doherty Earth Observatory of Columbia University), Dr. Le Bras (scientist at the Woods Hole Oceanographic Institution) and Dr. Ludka (postdoctoral researcher at the Scripps Institution of Oceanography) share with *Nature Communications* their experiences bringing together the LGBTQ+ community at geoscience conferences, and offer advice for how other disciplines can do the same.

What is your position, background and current research focus?

Benjamin Keisling (he/him, BK): I am a postdoctoral fellow at Lamont-Doherty Earth Observatory. I am a white cisgender queer man who grew up in Portland, Oregon. I did an undergraduate degree in physics and went on to get my Ph.D. in Geosciences. My research focuses on how ice sheets in Greenland and Antarctica have responded to climate change in geologic history, to better contextualize ongoing changes at the poles and improve projections of future sea-level rise.

**Figure:**
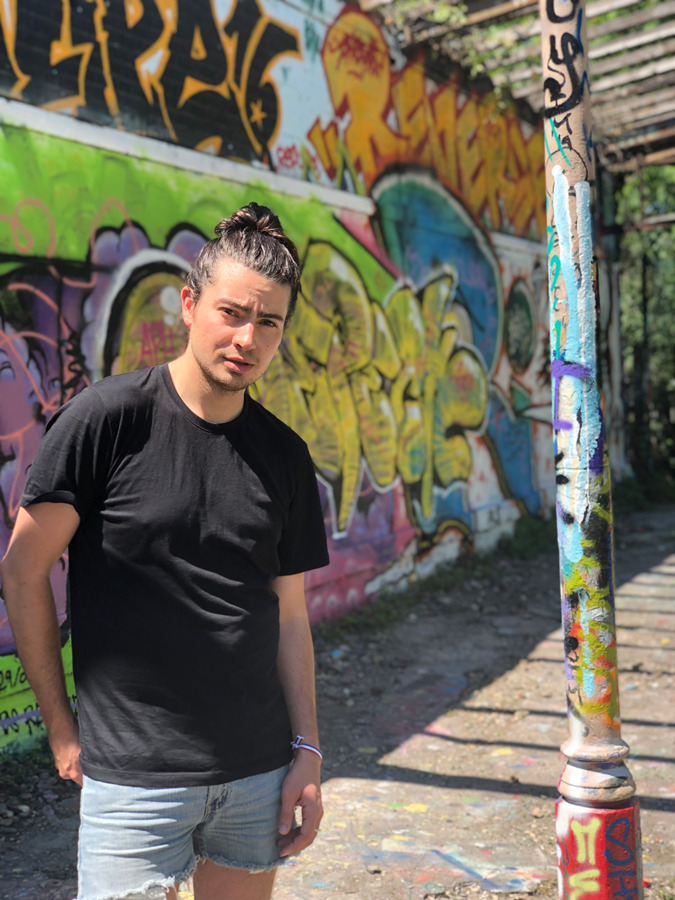
Dr. Benjamin Keisling, postdoctoral fellow, Lamont-Doherty Earth Observatory of Columbia University. Dr. Marine Cornuault

Isabela Le Bras (she/her, ILB): I am an observational physical oceanographer working as a tenure-track Assistant Scientist at the Woods Hole Oceanographic Institution (WHOI) interested in the physics of large ocean currents and their role in the climate system. My research has taken me from the warm Gulf Stream offshore of Cape Cod, where WHOI is located, to the icy Arctic Ocean. I have a B.A. in physics from UC Berkeley and a PhD in physical oceanography from the MIT-WHOI Joint Program. I am a half Mexican, half French, first-generation American queer woman who grew up on the West Coast of the US and Vienna, Austria.

**Figure:**
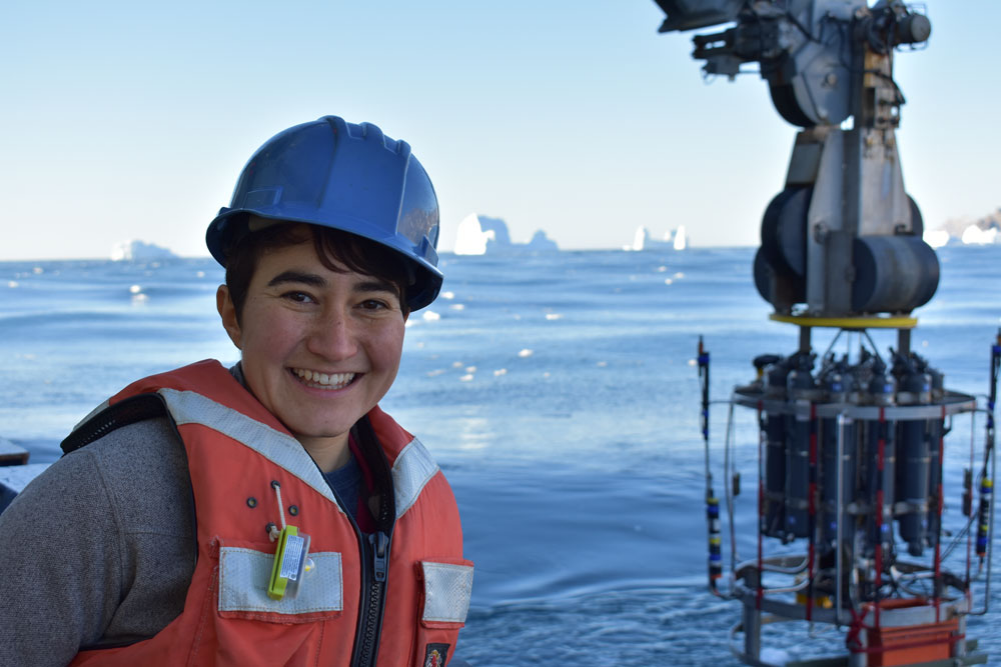
Dr. Isabela Le Bras, Assistant Scientist, Woods Hole Oceanographic Institution. EJ Rainville

Bonnie Ludka (she/her, BL): I’m a white queer/genderqueer woman who grew up stomping around streams and rivers near the Chesapeake Bay. I have a bachelor’s degree in physics and a PhD in physical oceanography. I’m currently a postdoctoral researcher at Scripps Institution of Oceanography (SIO). I have experience studying wave-driven beach erosion and recovery, assessing the use of beach nourishment (sand replenishment) as a coastal management practice, and developing new satellite techniques to map coral reefs. I am passionate about coastal research because it intersects many fields of fundamental science (physics, geology, ecology) and resilience-building with coastal communities.

**Figure:**
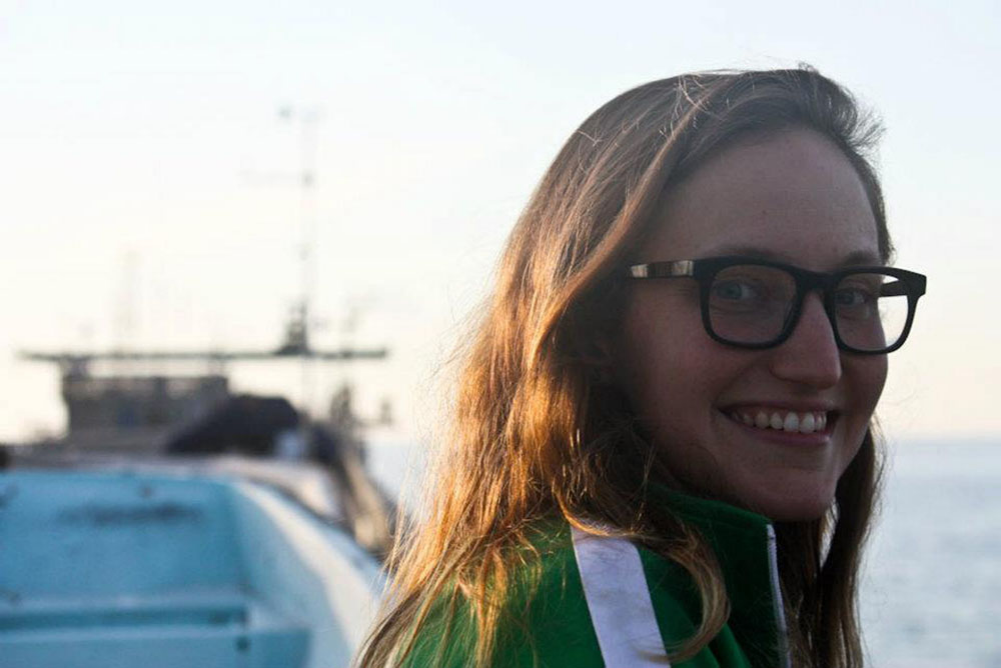
Dr. Bonnie Ludka, postdoctoral researcher, Scripps Institution of Oceanography. Dr. Nicholas Pizzo

What are, in your view, the biggest challenges for the LGBTQ+ community in the geosciences today?

**BK**: Homophobia and transphobia are the biggest challenges for the LGBTQ+ community in geoscience. There are certainly geoscience-specific barriers that are important to address as well, but I think we still have a huge gap in terms of who actually gets to participate in geoscience because of discrimination and harassment of LGBTQ+ people in geoscience, in academia, and in society at large.

Another challenge is the lack of data on sexual orientation and gender identity that could help us better understand how to support our LGBTQ+ colleagues. As geoscientists I think we share an appreciation for making decisions and testing hypotheses through data collection. At the same time, we don’t need comprehensive data to identify when (and where) harm is being done to LGBTQ+ geoscientists and to address that courageously and swiftly.

**ILB**: Feeling safe bringing your full self to work can be difficult because of homophobia and transphobia. This can range from the very real harassment and discrimination that Benjamin brought up to the exhaustion and pain that arises from (often unintentional) microaggressions. It’s much easier not to say anything than to constantly point out when a colleague has used a word or phrase that you find hurtful. I think this is particularly difficult for our trans and nonbinary community members. Educating the geosciences community and society at large to be sensitive to these issues without placing all the onus on the LGBTQ+ community to do the educating is a major challenge.

**BL**: I sometimes get asked, by well-meaning people, why I still work on LGBTQ+ issues, as if these issues don’t exist anymore. Yet, in the United States, where I live, discrimination against LGBTQ+ people is clear across all sorts of statistics. LGBTQ+ people experience more violence, houselessness, unemployment and uninsurance (healthcare) than their non-LGBTQ+ peers, where the numbers are particularly depressing/terrifying regarding Black trans people. These are problems that are not unique to the U.S. and can prevent LGBTQ+ people from attaining, maintaining, and succeeding in careers in geosciences. We have had some important recent legal wins for LGBTQ+ people in the U.S. but we still need many more. Of course, legal protection is only one part of how to dismantle homophobia and transphobia, but I think it highlights that we have both made a lot of progress and still have a long way to go.

Describe the genesis of creating the LGBTQ+ gatherings at the Ocean Sciences Meeting and the American Geophysical Union meeting--in particular, what motivated you to start these events, and how did you go about doing it?

**BK**: When I attended my first AGU in 2013 I remember searching “LGBT” in the meeting program, and finding only a single abstract. I attended my first “gAyGU” dinner at the AGU Fall Meeting in 2017. These events started as informal gatherings in the 1980s with about two dozen attendees, mostly gay white men - and despite efforts to get the event recognized as part of the official program, it had remained relatively clandestine until then. With the blessing of some of the long-time organizers, I reinvigorated that effort to get officially recognized so that we could reach more (particularly early-career) people. I created surveys, did a lot of tweeting, advocated through my position on AGU’s Diversity and Inclusion Advisory Board, and organized with past, present, and future leaders of the community to make LGBTQ+ programming at the Fall Meeting more visible and inclusive.Over the last couple of years a lot of what I have done is an attempt to ensure that future generations of geoscientists coming to that meeting find a robust representation not only of their scientific interests but also ways to connect with other geoscientists that share some of their identities and experiences.

**ILB**: From 2016 to 2020, I was the early career representative on the steering committee of MPOWIR (Mentoring Physical Oceanography Women to Increase Retention) when someone brought up whether MPOWIR should also be doing more to support the LGBTQ+ community in oceanography. As a member of the LGBTQ+ community I volunteered to lead this effort.

In graduate school I had been a part of GLOW, the LGBTQ+ affinity group in Woods Hole, which mainly revolved around social get-togethers a few times a year. What I valued most about GLOW was meeting queer folks who had been at WHOI for a long time and were out, proud, and thriving. Getting to know these role models through GLOW gave me the courage to be my full self at work and I wanted to help create that space for others, who may be the first or one of few LGBTQ+ folks at their institution. Hence the idea of a social get-together at our large bi-annual Ocean Sciences Meeting (OSM).

MPOWIR helped me conduct surveys, advertise, and coordinate logistics for an offsite dinnertime event during OSM 2018. I got connected with folks across the country leading similar efforts, including Benjamin, and tried to get advice and start building listservs. There was a lot of enthusiasm at our first event, but it was limited to my extended network and it was clear that we wanted future events to be advertised in the official conference materials and be more open to mingling. By the time OSM 2020 came around, Bonnie brought new energy to this effort and we pulled off a much larger, funded, event at the meeting venue.

**BL**: Because OSM 2020 was happening in San Diego, where I live and work, I somehow felt more inspired and responsible to act as a host, and specifically wanted to welcome my LGBTQ+ community to town. Isabela and I got in touch with others active in organizing LGBTQ+ events for the geoscience community at conferences, like Benjamin. I started learning about all the amazing work that had already been done in this regard over the last decades. I was shocked that I was somehow only just learning about gAyGU when I had been attending these conferences as an out queer person for over a decade! Through conversations with Isabela and Benjamin, I realized that we needed to have an event where attendance was not limited by word-of-mouth or word-of-social media. We decided it would be best to host our event onsite to ensure that it would be listed in the formal program and maximize accessibility and visibility. We envisioned a more open space rather than a sit-down dinner to better facilitate new connections across our community, and we called it the “Rainbow Reception for the LGBTQIA+ Community”.

Were there any challenges or roadblocks to launching these initiatives? If so, how did you overcome them?

**BK**: There have been a lot of challenges to building community among LGBTQ+ geoscientists, and there still are roadblocks we have yet to overcome. We have pushed relentlessly to get our annual dinner in the program, to have pronoun pins available, and to create a variety of LGBTQ+ networking spaces to serve the diverse needs of LGBTQ+ geoscientists. All of the challenges associated with these goals have been overcome through community building. The LGBTQ+ geoscience community is incredible and if I listed everyone who has helped to overcome these challenges it would use up the rest of the space allocated for this Q&A. I think there is no challenge that we can’t overcome if we continue to be as interconnected, brave, and resilient as we are now.

A challenge I think about a lot now is how we make the entire meeting or organization - not just the LGBTQ+ community events - affirming and positive experiences for everyone in our community. Not everyone has the privilege of attending a meeting and being able to purely focus on science or community building, because many people still have to invest energy in making sure they are safe during the meeting. “Safe AGU” is a program that directly tries to address this and I hope to see that grow in the future to protect geoscientists from kinds of harm that are not currently encompassed, like holding meetings in places that are not safe for the LGBTQ+ community, misgendering, and ensuring access to bathrooms for every geoscientist at the meeting.

**ILB**: I think another important thing to remember is that many (but certainly not all!) of the folks spearheading these efforts are early career scientists who also need to focus on building their careers. As institutions become more supportive of these types of efforts, I hope we can overcome the logistical challenges we have faced like getting affinity-group community events on the official conference calendar and getting funding, so that our time can be spent actually building community and on doing science!

**BL**: We wanted to schedule our event during a time that was not competing with conference presentations, which meant we would be hosting during a time when people would be hungry. We needed to feed people if we wanted them to come and stay. That’s when we started writing emails to Deans and Diversity Coordinators of ocean science institutions around the country to raise funds for hors d’oeuvres. Coordinating the catering, fundraising and the transfer of funds was a huge job for someone who has a full time job doing science. I hope that our institutions see how necessary these events are for our community and are able to incorporate them into conference planning and budgeting.

What are the benefits of these targeted networking events? Can you describe any positive impacts or outcomes you’ve seen?

**BK**: One year I was on my way to the annual LGBTQ+ networking event (now called “AGQ” to be more inclusive) and someone I had never met before came up to me and asked if that’s where I was going, and if they could walk with me. On the way, they told me about how it was the first time they had gone to something like that, how they weren’t out to anyone in their own geoscience community and how nervous and excited they were. I ended up running into that same person a few years later and they told me how it had meant a lot to them to find a community of geoscientists where they could safely be “out”. There are so many geoscientists who still cannot be themselves in the environments where they live and work, and if we can provide a place where they can be seen, be themselves, make friends, have fun - that is the outcome I am most interested in pursuing, and that is what drives me to do the work.

**ILB**: I had similar experiences of folks feeling comfortable being out for the first time at our OSM 2020 event, and I agree that this type of space can help people on their journey towards coming out and feeling more comfortable in their work environments. I feel very privileged to be comfortably out at work, but seeing so many LGBTQ+ oceanographers gathered together brought an unexpected and overwhelming wave of joy over me. Many generations of oceanographers were represented, and many important mentorship relationships have blossomed out of these events.

**BL**: When the Rainbow Reception finally happened, we had over 300 people show up. Like Isabela, when I saw my fabulous diverse community come together, where we could all just be ourselves without hesitation, I was overwhelmed with joy. I actually cried some tears of happiness (and probably exhaustion). The stories of connections made there are countless. The meaning of those connections ranges from inspiring people to stay in science or coming out at work, to rejuvenating those of us who are fatigued by being out in a society where we feel othered.

Do you have any advice for others who are trying to spearhead similar efforts in their own fields?

**BK**: Honor the past - we can learn a lot from the experiences of our predecessors - and at the same time allow yourself to envision or implement things in new ways. Think proactively about creating spaces that center the needs of the most marginalized and precarious members of the community. Ask others who have been building community before you for their help and support, and work with them to have the broadest possible reach and to create an inclusive community.

**ILB**: Advertise as widely as possible, preferably through the official conference material, include food, and make sure there is room for walking around and mingling. Welcome people as they arrive, especially if they are alone. Someone volunteered to host a transgender space within the larger event, which I think was important and successful.

**BL**: Follow Benjamin and Isabela’s advice. Look to your community for guidance, especially those who have identities different from your own. Ask for help getting things done. Make sure to take the time to enjoy your own event. Ask for feedback both before and after. Celebrate successes and work towards improvement by passing on what you learned. Let others morph your creations into something new.

What is the future of these events, or what are the next steps moving forward?

**BK**: For me, the next steps moving forward have to do with making the LGBTQ+ geoscience community inclusive and affirming for our trans and nonbinary community members and LGBTQ+ geoscientists of color. Historically these are groups that have not been centered in our community building, and that needs to change. There is tremendous opportunity to build solidarity among affinity groups that serve different populations, and in order to do that work effectively we have to ensure that our community is meeting the needs of geoscientists who experience marginalization along multiple axes of their identity (e.g. race, gender and disability status). I already see a lot of leadership and vision coming from the LGBTQ+ geoscience community in this regard and I am excited to see where the next generation of LGBTQ+ geoscience leaders will lead us.

**ILB**: I would love to see these LGBTQ+ community building events become standard parts of the program, as well as affinity events for other communities. I don’t think we are quite there yet, but I think it is an achievable short-term goal. I think we also need to be proactive in recruiting LGBTQ+ people of color and other underrepresented voices into the geosciences. The LGBTQ+ community has a role to play as allies as well!

**BL**: The OSM Rainbow Reception was built upon the work of so many events that came before it. Likewise, I’m hopeful that the Rainbow Reception will live on and transform. At each meeting it will look different, changing with our evolving community, and constantly improving to be as inclusive and meaningful as possible. For example, the name may change soon to be more inclusive, similar to how gAyGU is now AGQ. The more voices we have shaping these events the better, especially from the most marginalized in our community. We need to seek out these voices by doing our homework to create more inclusive spaces and connecting through genuine relationships rather than burdensome requests. As we work to make these events more inclusive, more of these meaningful connections can be made, and we build something better and better. Like Benjamin, I already see so much new wisdom and energy pouring into planning these events. With more institutional support, like having our events incorporated into standard conference programming, organization and budgeting, our impact could be enormous.

*The interview was conducted by Senior Editor Dr. Kyle Frischkorn.*

